# Stimulated cervical afferent input increases postural instability in older people with chronic neck pain: a cross-sectional study

**DOI:** 10.1186/s12877-024-04695-x

**Published:** 2024-02-14

**Authors:** Korawat Phapatarinan, Munlika Sremakaew, Sureeporn Uthaikhup

**Affiliations:** Department of Physical Therapy, Faculty of Associated Medical Sciences, Chiang Mai University, 50200 Chiang Mai, Thailand

**Keywords:** Cervical afferent, Balance, Older, Neck pain, Postural sway

## Abstract

**Background:**

Several potential causes can impair balance in older people. The neck torsion maneuver may be useful in demonstrating impaired balance caused by the stimulation of cervical proprioceptive input. Whereas evidence suggests impaired standing balance in older people with chronic neck pain, balance impairment during the neck torsion position and its relationship with clinical characteristics have not yet been investigated in this population. The aims of this study were to investigate whether the neck torsion position could significantly influence balance responses in older people with chronic non-specific neck pain and to determine the relationships between the balance responses and characteristics of neck pain.

**Methods:**

Sixty-eight older people (34 with chronic non-specific neck pain and 34 controls) participated in the study. Balance was tested using a force plate during comfortable stance with eyes open under four conditions: neutral head on a firm surface, neutral head on a soft surface, neck torsion to left and right on a firm surface and neck torsion to left and right on a soft surface. Balance outcomes were anterior-posterior (AP) and medial-lateral (ML) displacements, sway area and velocity. Characteristics of neck pain were intensity, duration and disability.

**Results:**

Overall, the neck pain group exhibited greater AP and ML displacements, sway area and velocity in the neck torsion position on firm and soft surfaces compared to controls (partial eta squared (η²p) = 0.06–0.15, *p* < 0.05). The neck pain group also had greater AP displacement, sway area and velocity in the neutral position on a soft surface compared to controls (η²*p* = 0.09–0.16, *p* < 0.05). For both groups, the neck torsion position displayed overall greater postural sway compared to the neutral position (η²p *=* 0.16–0.69, *p* < 0.05). There were no relationships between the postural sway outcomes and characteristics of neck pain (*p* > 0.05).

**Conclusion:**

The neck torsion maneuver, stimulating the receptors resulted in increased postural sway in older people, with a more pronounced effect in those with neck pain. The study provides evidence supporting the use of neck torsion for assessing impaired balance related to abnormal cervical input in older people with chronic non-specific neck pain.

## Background

While aging is associated with declining balance [1, 2], evidence suggests that neck pain is also related to impaired balance in older people [3–5]. The cervical spine has a highly developed proprioceptive system, which works together with the vestibular and visual systems to maintain posture and balance [6, 7]. Previous studies demonstrated that older people with neck pain had increased postural sway during standing compared to those without neck pain [3, 4], suggesting that the presence of neck pain was associated with a decline in postural stability over and above what is expected with normal ageing. However, the magnitude of altered proprioceptive input caused by neck pain remains unclear, considering that several factors including age-related changes, such as presbycusis, macular degeneration, impaired visual acuity and vestibular dysfunction, can also impair standing balance in older people.

It has been suggested that the neck torsion maneuver stimulates the cervical afferent receptors, but not the vestibular receptors [8, 9]. If performance in a torsioned position is worse than in a neutral position, it strongly suggests a cervical afferent influence. A study found that young patients with chronic neck pain had greater increases in postural sway with neck torsion compared to patients with unilateral vestibular loss and asymptomatic controls [9]. A recent study has also demonstrated the relationship between neck position sense and balance in the neck torsion maneuver [10], which supports a proposed mechanism of cervical-driven postural balance deficits. Thus, the neck torsion maneuver may further identify altered cervical afferent causes of impaired standing balance in older people with chronic neck pain.

The aim of this study was to investigate postural sway during standing in the neutral and neck torsion positions between older people with and without chronic neck pain in order to determine the impact of altered cervical afferent input on balance responses. The relationships between postural sway and characteristics of neck pain (i.e., intensity, disability and duration) were also explored in the study.

We hypothesized that increased postural sway during standing in the neck torsion position would be demonstrated in older people with neck pain compared to controls and when compared to the neutral position. Postural sway observed would also be positively correlated with some clinical characteristics of neck pain.

## Subjects and methods

### Participants

The sample size for the study was calculated based on mixed model design (within-between interactions), a power of 0.8 and a medium effect size (Cohen’s f) of 0.3, using G*Power 3.1.9.4. A total sample of 68 participants (34 in each group) was required.

Sixty-eight older people (34 with non-specific neck pain and 34 without neck pain) aged 60 years or older were recruited from local hospitals, physical therapy clinics and/or the community by advertising through posters and social media (e.g., Facebook, Line and Instagram). Nonspecific neck pain was defined as unidentified pathoanatomical cause [11]. Inclusion criteria for the neck pain group were chronic neck pain (≥ 3 months), an average pain intensity of ≥ 3 on a 0–10 cm Visual Analogue Scale (VAS) and a current Neck Disability Index (NDI) score of at least 10/100 [12]. The control group had no history of neck pain and headache for at least 6 months. Participants were excluded if they had a previous history of head and cervical spine injury or surgery, cervical range of motion in rotation to either left or right side < 45 degrees [8], any musculoskeletal problems that could affect balance (e.g., back pain and lower limb pain), known or suspected vestibular conditions (e.g., BPPV or Meniere’s disease), known or suspected visual problems (e.g., visual neglect and double vision), neurological problems that could affect balance (e.g., stroke and Parkinson’s disease), cognitive disorders (e.g., dementia and Alzheimer’s disease), having peripheral neuropathy (e.g., diabetes mellitus) and taking polypharmacy (≥ 4 types) or psychotropic medications [13].

### Clinical characteristics of neck pain

A questionnaire was administered to collect demographic data and relevant clinical characteristics (e.g., pain side and duration). A 0–10 VAS was used to assess pain intensity. The VAS is both valid and reliable with higher scores indicating a higher level of pain [14]. The NDI was used to quantify self-perceived disability associated with neck pain. The NDI has been shown to be a valid and reliable measure of neck disability [12, 15]. A higher percentage score indicates greater disability.

### Standing balance

A 40 cm × 60 cm stable computerized force plate (Model BTS P-6000; BTS Bioengineering Corporation, Quincy, MA) was used to assess postural sway during the standing balance test. The force signals from the force plate were converted from analog to digital, at a sampling rate of 100 Hz. A SMART-Clinic software and sway program (BTS Bioengineering Corporation, Quincy, MA) were used to analyze postural sway. Postural sway was characterized as displacement in the anterior-posterior (AP) and medial-lateral (ML) directions, sway area and velocity. The AP and ML displacements are determined based on the displacements of the center of pressure (COP) in the sagittal and coronal planes, respectively. The sway area represents the surface area covered by the COP during the movement. Velocity is obtained by dividing the COP excursion length by the trial duration. The mean value for each condition was used for analysis. In this study, the reliability of the measurement was good to excellent (ICCs range = 0.75–0.97).

Standing balance was measured with bare feet in a comfortable stance (feet about shoulder-width apart) in four different standing conditions: (1) neutral - eyes open on a firm surface (EOF), (2) neutral - eyes open on a soft surface (EOS), (3) neck torsion to the left and right - eyes open on a firm surface (EOF-torsion) and (4) neck torsion to the left and right - eyes open on a soft surface (EOS-torsion). For the neutral condition, participants stood with their feet in the straight-ahead position and the neck in a neutral position. For the neck torsion condition, the participant’s head was held in a neutral position with body and feet turned 45 degrees (marked on a force plate) to either side (left or right) [8, 9]. One examiner maintained the participant’s head position and another examiner assisted in repositioning the participant’s feet verbally and manually. Each condition was performed for 30 s two times [16, 17]. A two-minute interval was given between conditions. During the tests, all participants were instructed to maintain the position and stand as steadily as possible with arms by their sides. No manual contact was given by the examiners. Two attempts were allowed for each condition. Participants were withdrawn if they could not complete the tests. Standing balance was conducted in a quiet room. The examiners were blinded to participants’ pain conditions and were not allowed to question them about their pain conditions. Participants were requested to refrain from taking any medication that may influence balance for at least 6 h before testing.

### Statistical analysis

Independent t-test and chi-square were used to describe any differences in participant demographic data and characteristics of neck pain between groups. The Shapiro-Wilk test was used to determine normality of data. Paired-sample t-test was preliminarily used to test the differences in postural sway parameters between sides (left and right) in the neck torsion condition on firm and soft surfaces. No significant differences between sides were observed, thus the average values of both sides were used for further analysis of the neck torsion conditions.

Mixed model analysis of variance (ANOVA) was used to analyze within- and between- group differences in postural sway parameters (AP and ML displacements, sway area and velocity) for each condition. Where significant main or interaction effects were found, Bonferroni post-hoc tests were conducted for multiple comparisons. Effect size was calculated as the partial eta squared (η²p) and interpreted as follows: 0.01–0.05 small, 0.06–0.13 medium and ≥ 0.14 large [18]. The relationships between postural sway and pain features (intensity, disability and duration) for each condition were analyzed using Pearson’s correlation coefficient. The correlation coefficient values were interpreted as follows: 0.00–0.09 negligible, 0.10–0.39 weak, 0.40–0.69 moderate, 0.70–0.89 strong and 0.90–1.00 very strong [19]. A significance level was set at *p* < 0.05.

## Results

### Participants

Demographic data and clinical characteristics of the neck pain and control groups are presented in Table 1. There were no differences in gender, age and BMI between groups (*p* > 0.05). About 55.88% of participants with neck pain reported bilateral neck pain and 44.12% reported unilateral neck pain.

**Table 1:** Participants’ demographics and clinical characteristics related to neck pain

	Neck pain (*n* = 34)	Controls (*n* = 34)
Demographic data		
Gender (female, n)	31	31
Age (yrs.)	68.29 ± 0.92	68.12 ± 0.75
BMI (kg/m²)	23.63 ± 0.58	23.87 ± 0.51
Characteristics of neck pain		
Intensity (VAS, 0–10)	4.57 ± 0.24	-
Disability (NDI, 0–100)	21.88 ± 1.75	-
Duration (yrs.)	2.13 ± 0.44	-
Sides of neck pain (n)		
Unilateral (right, left)	9, 6	-
Bilateral	19	-

Data are presented as mean ± SD unless otherwise indicated

BMI = Body Mass Index, VAS = Visual Analog Scale, NDI = Neck Disability Index

### Effects of neck pain on postural sway

**Fig. 1:**
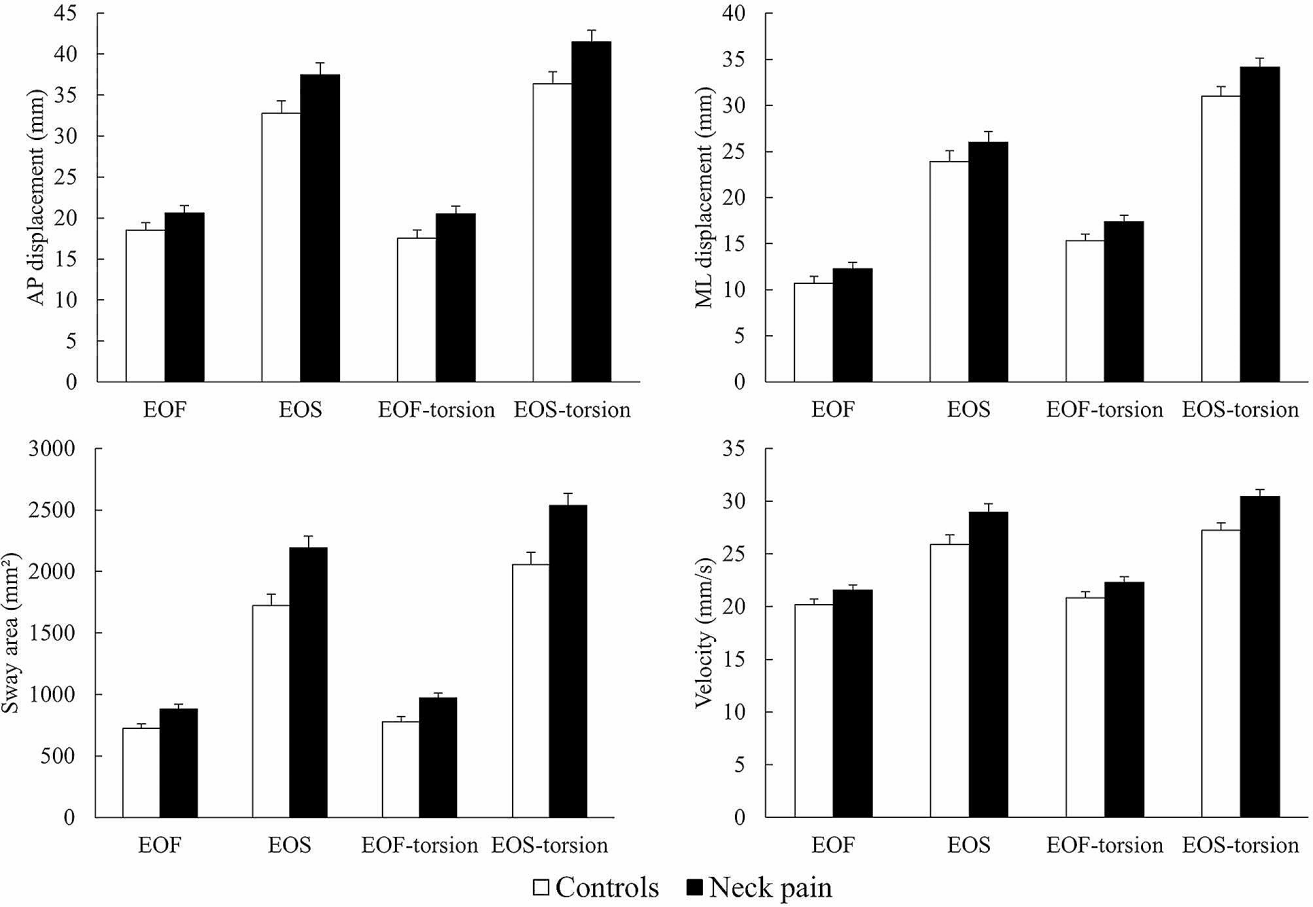
The means and standard errors for displacement in the anterior-posterior (AP) and medial-lateral (ML) directions, sway area and velocity in all standing balance conditions; EOF = eyes open on a firm surface; EOS = eyes open on a soft surface; EOF-torsion = eyes open on a firm surface with neck torsion; EOS-torsion = eyes open on a soft surface with neck torsion

All participants completed the balance tests and the neck torsion position did not significantly intensify their pain. The displacement (AP and ML directions), sway area and velocity in all standing balance conditions for the neck pain and control groups are shown in Fig. 1. The mean differences and 95% confidence intervals (CIs) of the postural sway parameters between groups are presented in Table 2. Compared to controls, the neck pain group had increased AP displacement in the EOS, EOS-torsion and EOF-torsion conditions (*p* < 0.05, η²*p* = 0.08–0.11) and increased ML displacement in EOF-torsion and EOS-torsion conditions (*p* < 0.05, η²*p* = 0.06 and 0.07, respectively). The neck pain group also had increased sway area in all conditions (*p* < 0.01, η²*p* = 0.13–0.16) and increased velocity in the EOS and EOS-torsion conditions (*p* < 0.05, η²*p* = 0.09 and *p* < 0.01, η²*p* = 0.15, respectively).

**Table 2:** The mean between-group differences (95% CI) in the postural sway parameters for all test conditions

		Main effect		Interaction effect	Neck pain vs. Controls	p-value
		Group	Condition	(Group x Condition)	Mean difference (95% CI)	
AP displacement (mm)		**0.01**	**< 0.001**	0.32		
	EOF				2.08 (-0.47–4.63)	0.11
	EOS				4.70 (0.54–8.86)	**0.03**
	EOF-torsion				2.97 (0.25–5.70)	**0.03**
	EOS-torsion				5.11 (1.00–9.22)	**0.02**
ML displacement (mm)		**0.03**	**< 0.001**	0.69		
	EOF				1.58 (-0.47–3.62)	0.13
	EOS				2.13 (-1.16–5.42)	0.20
	EOF-torsion				2.06 (0.07–4.06)	**0.04**
	EOS-torsion				3.12 (0.33–5.92)	**0.03**
Sway area (mm²)		**< 0.001**	**< 0.001**	**0.01**		
	EOF				159.26 (55.57–262.96)	**0.003**
	EOS				470.39 (207.83–732.95)	**0.001**
	EOF-torsion				193.36 (80.54–306.18)	**0.001**
	EOS-torsion				478.60 (202.35–754.85)	**0.001**
Velocity (mm/s)		**0.01**	**< 0.001**	**0.03**		
	EOF				1.37 (-0.11–2.84)	0.07
	EOS				3.02 (0.59–5.46)	**0.02**
	EOF-torsion				1.44 (-0.08–2.96)	0.06
	EOS-torsion				3.18 (1.26–5.10)	**0.002**

AP displacement = anterior-posterior displacement, ML displacement = medial-lateral displacement, EOF = eyes open on a firm surface, EOS = eyes open on a soft surface, EOF-torsion = eyes open on a firm surface with neck torsion, EOS-torsion = eyes open on a soft surface with neck torsion

### Effects of neck torsion on postural sway

The mean differences and 95% CIs of the postural sway parameters between conditions (neck torsion vs. neutral) for each group are shown in Table 3. Increased postural sway (displacement, sway area and velocity) was demonstrated in the torsion condition (both on firm and soft surfaces) compared to the neutral condition for both groups, (*p* < 0.05, η²*p* = 0.16–0.61 for the neck pain group and η²*p* = 0.17–0.69 for the control group). The exception was the AP displacement on a firm surface for both groups and the sway area on a firm surface for the control group (*p* > 0.05).

**Table 3:** The mean within-group difference (95% CI) in the postural sway parameters between the neck torsion and neutral positions

		Neck pain	p-value	Controls	p-value
		Mean difference (95% CI)		Mean difference (95% CI)	
AP displacement (mm)	EOF-torsion - EOF	-0.09 (-1.77–1.60)	0.92	-0.98 (-2.73–0.77)	0.27
	EOS-torsion - EOS	3.99 (1.21–6.77)	**0.01**	3.58 (0.69–6.47)	**0.02**
ML displacement (mm)	EOF-torsion - EOF	5.12 (3.75–6.49)	**< 0.001**	4.63 (3.24–6.02)	**< 0.001**
	EOS-torsion - EOS	8.13 (6.10–10.16)	**< 0.001**	7.14 (5.08–9.19)	**< 0.001**
Sway area (mm²)	EOF-torsion - EOF	89.18 (10.19–168.17)	**0.03**	55.08 (-23.91–134.07)	0.17
	EOS-torsion - EOS	343.10 (159.51–526.69)	**< 0.001**	334.89 (151.30–518.48)	**0.001**
Velocity (mm/s)	EOF-torsion - EOF	0.72 (0.26–1.17)	**0.003**	0.65 (0.18–1.12)	**0.01**
	EOS-torsion - EOS	1.51 (0.48–2.54)	**0.01**	1.36 (0.30–2.42)	**0.01**

AP displacement = anterior-posterior displacement, ML displacement = medial-lateral displacement, EOF = eyes open on a firm surface, EOS = eyes open on a soft surface, EOF-torsion = eyes open on a firm surface with neck torsion, EOS-torsion = eyes open on a soft surface with neck torsion

### Associations between characteristics of neck pain and postural sway

There were no associations between any characteristics of pain and postural sway outcomes (displacement, sway area and velocity) for all test conditions (*r* = 0.01–0.26, all *p* > 0.05).

## Discussion

This study determined the effect of neck torsion maneuver stimulating the cervical receptors on postural sway parameters (AP and ML displacements, sway area and velocity) during standing balance between older people with and without non-specific neck pain. The overall results revealed that the neck torsion position led to increased postural instability in older people, with a more pronounced effect observed in those with neck pain, reflecting postural control is more dependent on altered cervical afferents. The results also confirm previous findings suggesting decreased postural stability beyond the normal age-related changes in older people with neck pain [3, 4].

When considering postural responses to the neck torsion maneuver, the magnitude of the increased COP excursions (i.e., sway displacement, sway areas and velocity) was significantly greater in older people presenting with neck pain. Medium to large effect sizes (η²p ranging from 0.06 to 0.15) were also observed, suggesting a substantial difference in the COP excursions between the neck pain and control groups. This indicates the clinical significance of the neck torsion maneuver in identifying abnormal cervical afferent input as an underlying cause of balance impairments in older people with chronic non-specific neck pain. The results of this study are consistent with previous studies demonstrating the neck torsion maneuver resulted in greater postural deficits in patients with neck pain [8, 9]. Increased COP excursions were also observed in neck torsion position compared to neutral position, regardless of the presence of neck pain. The AP body sway has been proposed to be associated with self-reported musculoskeletal pain [20–22], whereas the ML body sway seems to underlie age-related changes [23, 24]. In the present study, it was noted that the increased COP excursions were displayed in both AP and ML directions, which is inconsistent with a previous study reporting in younger patients with neck pain that the effect of neck torsion was only seen in the AP direction [8]. The cervical muscles have a high density of muscle spindles, which are important for postural control [7]. It has been suggested that pain in the neck can alter cervical proprioceptive afferent [6, 7] and compensation strategies associated with proprioception deficits seem to lie within the proprioceptive system rather than overweighing of other sources of sensory afferents [25]. This may lead to increased postural sway, particularly in the AP direction in patients with neck pain. The neck torsion position further heightens inaccurate proprioceptive information, resulting in a greater increase in AP postural sway [8]. However, in older populations, pre-existing deficits in the sensorimotor system (e.g., vestibular and visual subsystems) [26–28] and proprioceptive deficits [7, 29] associated with age-related changes can also contribute to postural stability, in addition to the effects of neck pain and neck torsion position. Thus, this could be a reason for the increased postural sway observed in both AP and ML directions in older people with neck pain.

Consistent with previous findings [30, 31], the increased postural sway was greater on a soft surface than a firm surface for both groups, indicating surface firmness affects balance control. It is known that a soft surface diminishes somatosensory feedback and the effectiveness of ankle strategy [32, 33]. Postural instability, which is more evident when older adults are exposed to unstable surfaces, could be a strategy for optimizing the performance of postural control and reducing the risk of falling [34]. It has been suggested that increased velocity represents increased control activities and is related to age-related changes [35]. Increased velocity is also greater when the complexity of the task increased (e.g., standing on foam, standing with eyes closed and semi-tandem stance) [36]. The results of this study support and suggest that older people with neck pain, when subjected to tasks with different levels of complexity (i.e., standing on a soft surface and with neck torsion) had greater difficulties in performing the task, which were identified by increased velocity and sway (displacement and area). Considering challenging balance tests is an essential component of a fall prevention program for older people.

There were no associations between postural sway and neck pain features (intensity, disability and duration) during comfortable stance with eyes open in older people with neck pain, which are similar to previous studies conducted during narrow stance with eyes open and eyes closed in younger patients with neck pain [37, 38]. Increased postural stability may be independent of neck pain features. However, one study found the relationships between postural sway (velocity and sway area) during narrow stance with eyes closed and self-reported pain scores in younger patients with neck pain [39]. The discrepancy between the results may be attributed to variations in participants’ characteristics. It was noted that the levels of pain intensity and disability in our study were mild and the variability of the data was small. Further investigation into this matter is still warranted.

The overall results indicated that standing in the neck torsion position resulted in greater balance impairment compared to the neutral position in older people, with a more pronounced effect in those with neck pain. The neck torsion maneuver, which stimulates the cervical receptors, can be used as a specific test for assessing impaired standing balance attributed to altered cervical proprioception in older people with neck pain. This can also assist in developing strategies for managing and improving standing balance in this population. However, some limitations of this study must be addressed. Most participants in this study were female, which may limit the generalization of the findings. Other deficits related to age-related changes were not measured in this study. A larger population, including both genders, with a broader range of pain intensity, disability and duration should be investigated in future research. The role of proprioception in postural control in older people with neck pain should also be further explored. Additionally, future research should determine if neck pain is related to the risk of falls in older people.

## Conclusion

The results of this study demonstrated that the neck torsion maneuver results in increased postural sway (sway displacement, sway area and velocity) in older people, with a more pronounced effect in those with neck pain. The increased postural sway was observed in both AP and ML directions and when the complexity of the standing balance task increased. The increased postural sway was not correlated with clinical features of neck pain. It is worthwhile to use the torsion position for the assessment of balance impairment related to abnormal cervical input in older people.

**Declarations**.

## Data Availability

The datasets used and/or analysed during the current study are available from the corresponding author upon reasonable request.

## Abbreviations

AP: Anterior-posterior
ML: Medial-lateral
VAS: Visual Analogue Scale
NDI: Neck Disability Index
COP: Center of pressure
EOF: Eyes open on a firm surface
EOS: Eyes open on a soft surface
EOF-torsion: Eyes open on a firm surface with neck torsion
EOS-torsion: Eyes open on a soft surface with neck torsion
